# Coaches’ Criteria for Talent Identification of Youth Male Soccer Players

**DOI:** 10.3390/sports10020014

**Published:** 2022-01-18

**Authors:** Jan Fuhre, Arild Øygard, Stig Arve Sæther

**Affiliations:** Department of Sociology and Political Science, Norwegian University of Science and Technology (NTNU), 7491 Trondheim, Norway; jan_fuhre@hotmail.com (J.F.); arild-oygard@hotmail.com (A.Ø.)

**Keywords:** talent development, TID, relative age effect

## Abstract

Introduction: The main aim of this study was to examine which criteria coaches considered in the talent identification of youth male soccer players aged 13–16. The second aim was to describe how the coaches considered these criteria when identifying players for their club or regional teams and how these criteria take the impact of the relative age effect into consideration. Methods: We conducted qualitative, semi-structured interviews with six male coaches from a professional club academy or a regional team within the Norwegian Football Federation. Results: In line with earlier research, the results showed that the coaches considered the technical, tactical, and mental factors as the most important in talent identification. Further in line with earlier research, they considered that the physiological and sociological factors were of secondary importance, while anthropometric measures were considered the least important. Regarding the relative age effect, the coaches were aware of the effect and its consequences, while few of them had ways to reduce the effect and its impact on their talent identification process. Even so, the coaches highlighted the importance of considering a holistic approach to talent identification. Conclusion: The results show similarities with earlier research, but there is still a need for more longitudinal studies that investigate criteria for talent identification in youth football.

## 1. Introduction

Talent identification (TID) in soccer involves identifying talented players with the prerequisites and potential to become a professional player based on specific criteria [1]. It is considered to be a complex process due to the many factors and skills that affect the performance in soccer, often considered dynamic, impacting each other, and influenced by exercise, which increases the complexity even further [2,3]. It has also traditionally been based on coaches or scouts watching players in matches or training contexts over time who judge their performance and future potential to reach the elite level [4]. The concept of talent has also been related to TID and has usually been understood and used on players who have an above-average level of ability within a domain [5]. This traditional approach is a process seldom based on objective criteria, but on the coach’s subjective perceptions of the ideal player, skillset, and/or potential, where previous experiences and intuition of the recruiter influence the assessment [6]. Such a subjective assessment practice has been demonstrated by earlier research to be the norm in professional soccer around the world [1,7,8,9] as well as a practice that can lead to repeated misjudgments and limited continuity in identifying talent [10].

Since the review paper by Williams and Reilly [11] and their model on potential predictors of talent in soccer, research has tried to elaborate on which of the predictors would be considered the most important. In 2000, they introduced the physical, physiological, sociological, and psychological predictors, while Williams et al. [1] reintroduced the potential predictors of adult high performance in soccer with four predictors: skills, physical, psychological, and social. In the meantime, research has highlighted several skills that have shown to be of high importance for later success at the senior level, on both the motorial and cognitive levels [1]. In general, technical performance characteristics, such as ball control, dribbling, and passing, have proven to be important predictors of future success in elite-level soccer [12,13]. Huijgen et al. [12] found that technique was a discriminating factor already in youth soccer and that it was a potential indicator of which players would later succeed at the elite level. The sport-specific perceptual-cognitive skills, such as tactical skills, game understanding, creativity, positioning, and orientation, have also proven to be valid predictors of future success in football [12,13,14]. When developing tactical skills and specifically perceptual–cognitive skills, the content of training and the learning environment that the players are exposed to have shown to have great influence [15,16,17]. When it comes to physiological factors, young elite athletes generally tend to score higher than their less skilled peers, which is in line with what one sees in senior football [18]. Physical characteristics and anthropometric proportions are factors that the practitioner has little or no influence on him/herself, such as height, growth, physique, and muscle composition [11]. Research has also identified psychological factors, such as self-confidence, resilience, concentration, commitment, discipline, adaptability, motivation, and the ability to cope with different challenges, as significant predictors of development and later success [19,20,21,22,23,24]. Furthermore, there seems to be an increasingly broad consensus that this is an area of importance and has gained more attention in both the identification and development of young talented players [25].

The identifiers in the process, the coaches, highlight technique as highly important among coaches and practitioners [6], especially related to ball control [26], dribbling, first touch, passing/shooting, and technique under pressure [6,27]. Perceptual–cognitive characteristics such as decision making, positioning, and game understanding have been highlighted as especially important by coaches and practitioners [6,26,28], while anthropometric, physiological, mental, and social factors have been deemed less important by coaches and practitioners [6,28]. However, in the recent study by Bergkamp et al. [26], the anthropometric and physiological factors were deemed as important as tactical skills by the participating scouts. The coach’s role in relation to the identification of talent is also related to the coach’s perception of skills that are important to succeed at the elite level and awareness of how they develop them most effectively [16,29]. Due to the complexity of soccer as a sport, there are many factors to consider [1]. Therefore, the research suggests that a holistic approach to identifying and developing talent, which includes technical factors, mental factors, physiological factors, and social factors, such as family relations, parental support, and training load, etc., may provide better insights and decisions when trying to identify talent [1,30,31].

An essential factor to consider when identifying and selecting players is the relative age effect (RAE), which is prominent when dividing players into chronological age groups with a span of one year [32], reinforcing the physical, cognitive, and emotional differences based on maturation [33]. However, there appears to be little or no difference in technical skill performance between early and late matured players [34]. This has been related to the compensation phenomenon [1], where players compensate for poor skills in one area with good skills in another to be able to compete with their lower maturation. This is reflected in the fact that academy players born in the last quarter have a higher chance than those born in the first quarter of achieving professional status in senior football in the end [35]. Studies have shown that football coaches usually do not take the RAE into account in the selection process, despite knowledge of the phenomenon [36].

The main aim of this study was to examine which criteria coaches considered in the TID of youth male soccer players aged 13–16. The second aim was to describe how the coaches considered these criteria when identifying players for their club or regional teams and how these criteria take the impact of the RAE into consideration.

## 2. Materials and Methods

In this study, we adopted a qualitative research design, using semi-structured interviews.

### 2.1. Participants

This study is based upon six semi-structured interviews with active male coaches in regional teams under the auspices of the Norwegian Football Federation and academy coaches connected to professional Norwegian soccer clubs. The coaches had a mean age of 34.16 (std 6.36), with an average of 6.66 (std 2.06) years’ experience as coaches. Two coaches had a UEFA-A license and the other four a UEFA-B license. Two of the coaches had a master’s degree in sport science, while one of the coaches had a bachelor’s degree in sport science.

### 2.2. Data Collection

The semi-structured interviews were conducted to gain further insight into the coaches’ perceptions of talent, TID, and the selection process. A formal interview guide was used to gain insight into the coaches’ perspective of talent, TID, identification criteria, and selection mechanisms. The interview guide was also open to relevant follow-up questions in relation to the coaches’ responses to the questions asked to gain relevant perspectives and insights from each of the participants. All interviews were individual and conducted digitally, using either Zoom or Microsoft Teams. The interviews were approximately one hour each and were transcribed in their full length by the interviewer.

### 2.3. Data Analysis

The interviews were audio-recorded and transcribed. To provide anonymity, the coaches were given identification codes, I1–I6 (Informant 1–Informant 6). Following the six steps suggested for qualitative analysis by Braun et al. [37], the following steps were used when analysing the data: (1) transcribing, reading, and re-reading the data; (2) generating initial codes, such as TID and perception of skills; (3) making use of deductive codes and identifying lower-order themes under the initial codes (e.g., ball control under technical skills), the data was coded by the second author and thereafter by author one and three with the intention to increase inter-rater reliabilities, and furthermore; (4) laying out the main topics from the data material with the use of ongoing member reflections; (5) reviewing the final categories and sub-themes; and (6) writing a report and presenting the data. Having processed all the data from the six interviews, we ended up with four central topics: perception of talent, talent development, talent identification, and relative age effect, reflecting the interview guide and presented in the results section. In the following presentation of the results from the six participants from the clubs and the local football federation (FA), I1, I5, and I6 represent region 1, and I2–I4 represent region 2.

## 3. Results

### 3.1. Coaches’ Perception of Talent

To get an impression of how coaches in the end identify talents, the coaches were asked about how they view talented players in soccer in a broader perspective. Mostly, the coaches said they viewed talent as someone who possessed abilities out of the ordinary. In addition, they point to talent as the potential to be a top-level athlete later in their career:

I3: There is someone who has special abilities, and it can be in football or music (…) and it is that you as a player have something more than what the others have.

One of the coaches, however, had quite a different view on talent when linking it with interest in the sport, the willingness to train, and feeling joy when practicing.

I2: No, it is a comprehensive concept, which deals with a lot. But for me, the talent is about the one who likes to play football the most and who is willing to go to great lengths to play football. It is in a way the first thing I think of as a talent concept, and then I know for myself that it is much more comprehensive than just that.

Overall, the coaches view talent as something extraordinary, regarding either performance or skills or regarding an extraordinary interest for the sport, which again leads to many accumulated hours of practice activity and joy when practicing the sport.

### 3.2. Talent Development: The Skills That Count

Asked about which skills characterise a talent in the age group of 13–16 years, the coaches varied a bit in their perceptions, but all of them somehow mentioned technical and tactical skills as the main characteristics of talent in this age group. Three of the coaches highlighted the technical and tactical aspects, such as first touch and decision making, as the most important skills when judging players’ potential and talent:

I4: Creativity and choices. I am interested in football players who have good close-up technique, good touch (…) I think it is very important to have a good deal of self-training with the ball (…) And it is now an experience I have had, that those who I have had, and whom I have seen are our best, creative players; they have spent a lot of time with the ball (…) so I look at skills, choice, execution, what you do with the ball rather than the physical.

The other three coaches also mention technical and tactical aspects as highly important, but they are also concerned with factors such as interest, motivation, drive, and the will to train as important characteristics in that specific age group, considering the ability to practice and, through practice, developing soccer-specific skills:

I3: You must have a presence, motivation, an inner drive. That is probably what is most important. And if you have it, then you usually have something else too, I think. And it must come from a young age; you must have played a lot with the ball since you were little. Technical and tactical, as I said, you can be very technical, but you lack everything else within the mental or the psychological aspect, like that you are not a nice person. Football is a team game, so it is clear to me that the psychological factors that we are talking about in a football context are very important.

Regarding how the coaches take their own skill valuation into account in their daily work with their players, all the coaches again point to the technical and tactical skills as the focal starting point for their coaching sessions. However, the two that get the most attention and the reasoning behind them differ among the coaches:

I3: (…) technique and tactics are a bit connected, but technique, call it basic skills, if you do not have it when you are 14 years old, then it limits the possibilities, I think. Tactical is like … you learn a lot through play and games at a younger age, and then it comes more and more into the 13–19 age group, and then it’s about cracking the codes quite quickly.

One of the coaches (I3) considers technical skills as the most important at an early stage for young players and considers tactical skills as something that comes later in the development process. This points to a fragmented understanding of the developmental process, such as developing one skill at a time. I1 also considered the technical skills as more important than the tactical skills but still pointed out that, in his club, the tactical skills tended to have more focus in the daily work:

I1: It is difficult to say that we look more at one or the other, but at the same time I can add that the club I am in has been very concerned with the decisions they make, the understanding of the game, and making good choices. And I may have been a little soft for it myself, which players make good choices. Then you can, to a certain extent, imagine that it is easier to develop (decision making) than the technical execution, so a slightly subjective answer to that (…).

### 3.3. Talent Identification

All the coaches in this study pointed out that their assessment of players’ talent and potential was based on subjective measures, and usually, a player’s performance in match situations was an essential factor:

I1: (…) The assessments we make on who we think fits and does not become subjective. And we are concerned with getting them into our context, to come in and train with us, to see if they can adapt to our tactics (…).

The coaches’ answers indicated some differences between the club and regional team coaches, where factors concluded as important by the Norwegian Football Federation were also highlighted by the regional teams’ coaches. The coaches at the club level were guided more by their club’s playing style and tried to identify players who would fit within that specific way of playing. Both, however, were still based on subjective measures and opinions.

Coach 2 coupled the definition of talent with interest and motivation towards the sport, which also influenced his perception of how early a talent could be identified:

I2: If you just take … that of finding a player who is genuinely interested in playing football, then you can see it quite early. We have a couple of examples from our own club. We have two players who were born in 2010 and are in their eleventh year, and you see they are genuinely interested in playing football. Whatever opportunity they have, they play football. And that is a starting point for something that can help them in later years. (…) They will lay an insanely good foundation in relation to the others, who are there only on team training, for example.

The reasoning behind this perception is that an extreme interest and joy in playing the sport itself will cumulate in more hours of practice on their own.

The coaches considered it possible, but challenging, to identify talented players quite early, especially if you use the players’ current abilities as the benchmark, even if they are 6, 10, or 13 years old. At the same time, they were clear about the complex nature of identifying talent regarding future potential as the following citations illustrate:

I1: (…) it is clear that a six-year-old can be much better than the other six-year-olds, and then he is better than the others at that time. And then the question is whether you can call it a talent or not. So, it’s probably easy to mix talent with the one who is good there and then, but I think more that it is aimed at the one who has potential. So, you can see if players are good already when they are six years old, but if it is a talent is very difficult to say, because it is impossible to say who will succeed.

I6: Basically, in my opinion, you see the football technical and coordinative abilities when they are 12–14 years of age, but then it differs even more when they are 16 years old. When you see the choices they make regarding school, you start to make some life choices (…).

Even if the coaches state that it is possible to identify the talented players quite early based on their current abilities, they are also very clear about the difference between current abilities, which may be better than others, and the future potential to reach the top level and develop the already good skills even further. Some also point out that different factors such as motivation, personal traits, and choices in life when they are old enough to make their own choices play a big role in determining the level of success a young player will have at the senior level. Those factors, according to the coaches, are harder to identify early, and they make a point out of differing between abilities and potential.

### 3.4. Relative Age Effect: The Consequence?

Identifying talented footballers at an early age often involves an impact from the RAE. The coaches in this study all answered that they knew about the concept of RAE and the potential challenges that it leads to. They answered that they do not consider physical skills or characteristics as important as the technical and tactical skills, which again plays a role when considering the RAE in their decisions. Even so, as Coach 5 pointed out, there is still an advantage if you are physically well-developed from an early age, because players’ abilities in real time often are higher, regarding obtaining results here and now. Coach 5 also made the point that the early-developed players may allow themselves bad habits technically and tactically because they can compensate with their physique.

I5: The advantage is that you are very efficient if you are well-developed physically. The disadvantage of this is that one then tends to acquire some bad habits, which they must unlearn when things level out more physically. In the same way, if one is very weak physically, then one can acquire some good habits to cope.

Another challenge related to selecting early developed players is that they will get more and better follow-up from coaches and clubs in their path, which, again, may lead to other talented players getting too little attention in their important development years.

When asked if they believe that talented players slip under the radar because of the RAE, the coaches partly disagreed, where some meant that they absolutely missed out on potential elite players, while others did not. Some of the arguments were that the deselected players potentially could give up too early or that the focus was on results, while some meant that the best players would always be identified:

I3: Yes, I think so. I think the players may not even reach their maximum level because they give up maybe a little early. And then we see that we may also be a little impatient not to give them the time they need to grow up then, as they call it.

I2: No, I do not think so. I think in a way that we can pick up those who can be the best or are the best; we will probably get them, I think. And then you can ask yourself the question of whether you might lose out on a broader group with potential to get to an OK Norwegian level. Maybe, but the best I think we will get.

## 4. Discussion

The main aim of this study was to examine which criteria coaches considered in the TID of youth male soccer players aged 13–16. The second aim was to describe how the coaches considered these criteria when identifying players for their club or regional teams and how these criteria take the impact of the RAE into consideration. The results indicated that the complexity of soccer makes it a very challenging task to identify the most talented players who will go on to succeed at the senior level from an early age [38]. These coaches, like earlier studies, show a high appreciation for the technical and tactical factors [11,30]. This also corresponds well with what has been seen in previous studies on coaches’ criteria for selection [6,28]. This also highlights the tradition of identifying and selecting talented players based on subjective criteria, which vary across cultures and clubs [1,6,7,9].

The coaches’ overall perspective is in line with earlier research’s definition of talent as someone who possesses abilities above average [8]. In particular, technical and tactical skills, such as ball control, dribbling, passing, creativity, positioning, and decision making, have proven to be valid predictors of future success [12,13,14]. Several of the coaches in this study also emphasised game understanding and positioning as important tactical qualities, which is in line with previous research in both qualitative studies [27,39,40] and quantitative studies [6,26,28]. This also relates to the complexity of judging talent, which corresponds well to earlier research on players who later succeed at the senior level [4]. The coaches also mention the player’s motivation, willingness to learn and train, and, maybe most importantly, the joy they get from playing the game as important factors. This differs a bit from the sport-specific focus of many coaches but nonetheless shows a broad understanding related to the complexity of identifying and nurturing talent. This also falls in line with research into traits and personalities who have reached the top level in their sports, which highlights the inner drive, discipline, and motivation of the succeeding athletes but also shows the differences between personalities, even if some traits are alike [19,21,22,23]. Earlier research has highlighted that interest, motivation, and joy lead to more self-organized training [41]. Part of the reasoning behind this perception is that an extreme interest and joy in playing the sport itself will cumulate into more hours of practice on their own, which research has shown to be a discriminating factor regarding later success at the elite level [41].

This study also found that the coaches, to a lesser degree, focused on the physiological attributes, which is also in line with earlier research and often related to the fact that these skills are trainable [42] and that differences may result from more systematic training over time [11]. Research has still shown that, for example, running velocity may be a useful predictor for later success at the elite level [12], but in general, one should still be careful about selecting talent based on physiological superiority. Another challenge related to selecting early developed players has been the access to better and more follow-up from coaches and clubs in their path, which, again, may lead to talented players receiving too little attention in their important development years, which studies have pointed to in the past [1,34,43]. This has also often been related to the RAE and the awareness of the effect, which have been found to be prominent in the TID process [4]. The coaches in this study, however, highlighted that the late-blooming players probably still could succeed later, even if they were not selected at the first crossroad. Even so, studies have pointed to the possible benefits one receives from early selection, which may reinforce the differences between players’ abilities because of better coaches and closer monitoring of their development [1]. Club coaches have this impression of players breaking through even if they are not selected because their potential and talent might be expected since they mostly work on a short-term basis. A valid point that the coaches make is that the differences between players, based on the RAE, are smaller when it comes down to technical and tactical skills, which they emphasise as most important in their view [12,32], and this may even lead to compensational development from the less physically developed players to be able to cope in games.

It is interesting to note that all the coaches seem to think that they can identify talent quite early based on players’ technical abilities here and now, even if they also point to the complexity of the development and especially the identification process. What supports the notion of being able to identify talents early on based on their technical abilities are the earlier findings that point to the technical skills as a potential early discriminator between elite and sub-elite players [12]. The technical skills are also less affected by the RAE, as we have seen earlier [12,32]. Even if the technical abilities might give an early indication, many coaches tend to mix the technical abilities and potential with early achievements, which, again, can stem from physical superiority and so forth.

### 4.1. Limitations and Methodological Issues

The small sample size must be considered as a limitation of this study. Furthermore, interviews with the use of teams have disadvantages because of the lack of closeness to the coaches interviewed. This might have impacted the results in the way that the participants might have misunderstood the questions or the reduced access to the interviewers’ body language might have been misunderstood or vice versa.

### 4.2. Future Studies

There is still a need for more longitudinal studies that investigate criteria for TID in youth football. Furthermore, studies comparing coaches with different roles, such as club coaches both at grassroot and elite levels and football federation coaches representing the national TID system, are needed. The TID process will most likely be impacted by the context in which the coaches are coaching, such as the degree of follow up and responsibility in everyday training, where club coaches have a much bigger impact on the players’ development compared to regional federation coaches in this study. Another vital question would be the background of the coaches related to both experience (years and level) and coach education. Knowledge and experiences from different levels (club, federation, school, etc.) might impact the coach’s perception on both talent development and the TID process. Matin and Sæther [44] found in a study on male and female football players in a sport specialization program (SSP) and elite sport specialization program (ESSP) in upper secondary schools in Norway that the players from the SSP school considered the school coach to focus significantly (<0.01) more on technical skills than the club coach. The players from the ESSP, however, considered the club coach to focus on technical skills as compared to the school coach. Furthermore, the players from the ESSP regarded the club coach to be significantly (<0.01) more focused on physical skills as compared to the players from the SSP. As discussed in the study, there are obvious reasons as to why coaches in different contexts (club vs. school) have different focuses in their training sessions and periods since only club coaches are responsible for a team, while school coaches are more naturally focused on individual physical and technical development.

Even though the intention of the present study was not to address the coaches’ background, which impacted the TID process, a vital question would be to address how their experience, education, and knowledge impacted the TID process. Earlier research has shown that coaches refer to “gut feeling” [40] and “practical sense” in the TID process. Peterson [45] has furthermore raised the question if football players succeed despite the talent selection system (the case of Sweden), both based on the prevalence of the RAE in the TID processes and the lack of precision in the TID criteria. He even questioned the idea of identifying talent at an early age based on the lack of success in most TID systems [2]. Future studies on perceptions of TID and RAE should also include the issue of bio-banding and how it impacts the coaches’ TID process—an issue raised in the newly published book by Kelly et al. [46].

Another issue future research should also address is the impact of how the national TID system and football federation impacts the coaches’ approach in the TID process. The coaches in this system may have an impact on each other’s approach in the TID process, with the risk of group thinking when the coaches discuss the players in the TID process.

## 5. Conclusions

This study has provided qualitative insight into the process of TID among youth soccer coaches in Norway. The results implied that the coaches considered the technical, tactical, and mental factors as the most important in TID—more specifically, ball control, decision making, and motivation (inner drive). They furthermore considered, in line with earlier research, that the physiological and sociological factors were of secondary importance, while the anthropometric measures were considered the least important. Regarding the RAE, the coaches were aware of the effect and its potential consequences, while few of them had ways to reduce the effect and its impact on their TID process. Even so, the coaches highlighted the importance of considering a holistic approach to TID, with all four potentially predicting categories of Williams et al.’s [1] model. There is still a need for more longitudinal studies that investigate the criteria for TID in youth football and studies comparing coaches with different roles in the TID system.

Key findings:

- Coaches considered the technical, tactical, and mental factors as the most important TID criteria.
- Despite awareness of the relative age effect and its potential consequences, few of them had ways to reduce the effect and its impact on the TID process.

The results show similarities with earlier research, still indicating a need for longitudinal studies that investigate criteria for TID in youth football.

## Data Availability

The data are not publicly available as data sharing was not included within the original study ethics submission or participant consent form.
